# Accelerated high-resolution free-breathing 3D whole-heart T_2_-prepared black-blood and bright-blood cardiovascular magnetic resonance

**DOI:** 10.1186/s12968-020-00691-3

**Published:** 2020-12-14

**Authors:** Teresa Correia, Giulia Ginami, Imran Rashid, Giovanna Nordio, Reza Hajhosseiny, Tevfik F. Ismail, Radhouene Neji, René M. Botnar, Claudia Prieto

**Affiliations:** 1 School of Biomedical Engineering and Imaging Sciences, King’s College London, Lambeth Wing, St Thomas’ Hospital, London, UK; 2 MR Research Collaborations, Siemens Healthcare Limited, Frimley, UK; 3 Escuela de Ingeniería, Pontificia Universidad Católica de Chile, Santiago, Chile

**Keywords:** Late gadolinium enhancement (LGE), Coronary magnetic resonance angiography (CMRA), Black-blood, Compressed sensing, Image navigator, Respiratory motion compensation

## Abstract

**Background:**

The free-breathing 3D whole-heart T_2_-prepared Bright-blood and black-blOOd phase SensiTive inversion recovery (BOOST) cardiovascular magnetic resonance (CMR) sequence was recently proposed for simultaneous bright-blood coronary CMR angiography and black-blood late gadolinium enhancement (LGE) imaging. This sequence enables simultaneous visualization of cardiac anatomy, coronary arteries and fibrosis. However, high-resolution (< 1.4 × 1.4 × 1.4 mm^3^) fully-sampled BOOST requires long acquisition times of ~ 20 min.

**Methods:**

In this work, we propose to extend a highly efficient respiratory-resolved motion-corrected reconstruction framework (XD-ORCCA) to T_2_-prepared BOOST to enable high-resolution 3D whole-heart coronary CMR angiography and black-blood LGE in a clinically feasible scan time. Twelve healthy subjects were imaged without contrast injection (pre-contrast BOOST) and 10 patients with suspected cardiovascular disease were imaged after contrast injection (post-contrast BOOST). A quantitative analysis software was used to compare accelerated pre-contrast BOOST against the fully-sampled counterpart (vessel sharpness and length of the left and right coronary arteries). Moreover, three cardiologists performed diagnostic image quality scoring for clinical 2D LGE and both bright- and black-blood 3D BOOST imaging using a 4-point scale (1–4, non-diagnostic–fully diagnostic). A two one-sided test of equivalence (TOST) was performed to compare the pre-contrast BOOST images. Nonparametric TOST was performed to compare post-contrast BOOST image quality scores.

**Results:**

The proposed method produces images from 3.8 × accelerated non-contrast-enhanced BOOST acquisitions with comparable vessel length and sharpness to those obtained from fully- sampled scans in healthy subjects. Moreover, in terms of visual grading, the 3D BOOST LGE datasets (median 4) and the clinical 2D counterpart (median 3.5) were found to be statistically equivalent (p < 0.05). In addition, bright-blood BOOST images allowed for visualization of the proximal and middle left anterior descending and right coronary sections with high diagnostic quality (mean score > 3.5).

**Conclusions:**

The proposed framework provides high‐resolution 3D whole-heart BOOST images from a single free-breathing acquisition in ~ 7 min.

## Background

Late gadolinium enhancement (LGE) cardiovascular magnetic resonance (CMR) imaging has emerged as the clinical reference standard for assessing viability following myocardial infarction [1, 2]. Moreover, LGE imaging is gaining importance in the diagnostic and prognostic evaluation of a range of heart muscle diseases where fibrosis can develop [3–7]. LGE imaging is performed after the administration of a gadolinium (Gd)-based contrast agent using inversion recovery (IR) prepared T_1_-weighted gradient echo sequences [1, 8–10]. The inversion time (TI) is set to null the signal of healthy myocardium in order to maximize the contrast between infarcted/fibrotic and normal myocardium. This method is highly dependent on the correct selection of TI for healthy myocardial signal nulling.

Phase-sensitive IR (PSIR) LGE is an alternative approach that is less sensitive to the TI selection [11, 12]. Conventional PSIR sequences require an IR-prepared image (or “magnitude image”) and a proton density weighted image (or “reference image”) to provide a reference for background phase. However, conventional PSIR sequences provide suboptimal contrast between scar tissue and the blood pool, meaning that sub-endocardial infarctions could be difficult to detect or delineate.

Black-blood PSIR LGE schemes have been proposed to improve scar-blood contrast by suppressing both the healthy myocardium and blood pool signal [13]. This can be achieved by combining an inversion pulse with a T_2_ preparation or magnetization transfer preparation [13–16]. Most existing PSIR LGE sequences are, however, limited to 2D acquisitions that are performed under a breath-hold. Recently, free-breathing 3D whole-heart PSIR acquisitions based on diaphragmatic navigator gating have been proposed [17, 18]. However, these approaches have low scan efficiency that lead to long and unpredictable acquisition times, and thus, may result in reduced image quality due to the change of the TI over time as well as disrupted workflows. In addition, PSIR sequences require the acquisition of a reference image, which doubles the scan time without providing additional diagnostic information. Recently, Ginami et al*. *proposed a free-breathing 3D whole-heart T_2_-prepared Bright-blood and black-blOOd phase SensiTive inversion recovery sequence (BOOST) for simultaneous bright-blood coronary angiography and black-blood LGE imaging [19]. With BOOST, magnitude and (bright-blood) reference whole heart datasets are acquired and combined in a PSIR reconstruction to obtain a third, complementary, black-blood volume. Additionally, BOOST uses image-based navigators (iNAVs) [20] to achieve 100% respiratory scan efficiency and predictable scan times. The T_2_-prepared BOOST sequence allows for visualization of myocardial infarction with excellent scar-blood contrast. In addition, the reference image provides a complementary bright-blood volume for cardiac anatomy and coronary lumen visualization (coronary CMR angiography, CCMRA). However, high spatial resolution T_2_-prepared BOOST (< 1.4 × 1.4 × 1.4 mm^3^) requires relatively long acquisition times of ~ 20 min. Yet, high-resolution 3D BOOST acquisitions are required to ensure accurate detection of scar location, size and transmurality, and identify coronary stenosis. This information is important to risk-stratify patients and guide revascularization decisions.

A non-contrast-enhanced version of BOOST has also shown potential for simultaneous bright- and black-blood whole-heart CMR for coronary lumen and thrombus visualization [21]. However, like post-contrast BOOST, high-resolution non-contrast-enhanced BOOST also requires long acquisition times.

Here, we propose to accelerate the T_2_-prepared BOOST sequence to enable high-resolution whole-heart 3D CCMRA and black-blood PSIR imaging in a clinically feasible scan time (< 10 min). This is achieved by extending XD-ORCCA (Optimized Respiratory resolved Cartesian CCMRA) [22], a highly efficient respiratory-resolved motion-corrected reconstruction framework, to BOOST imaging. XD-ORCCA reconstruction, which was originally introduced for fully-sampled 3D Cartesian CCMRA, exploits 2D translational motion information (extracted from iNAVs) to increase the sparsity in the respiratory dimension and to compensate for residual respiratory motion [22]. In this study, the feasibility of accelerating the T_2_-prepared 3D BOOST sequence [19, 21] using XD-ORCCA was tested in 10 patients with suspected cardiovascular disease, using an accelerated post-contrast BOOST sequence, at the end of a clinical CMR examination. Due to technical and ethical limitations, it is difficult to test new contrast-enhanced CMR sequences in patients and thus, the optimal acceleration factor employed was identified by first testing different acceleration factors using non-contrast-enhanced 3D BOOST acquisitions in 12 healthy subjects.

## Methods

### T2-prepared BOOST sequence

**Fig. 1:**
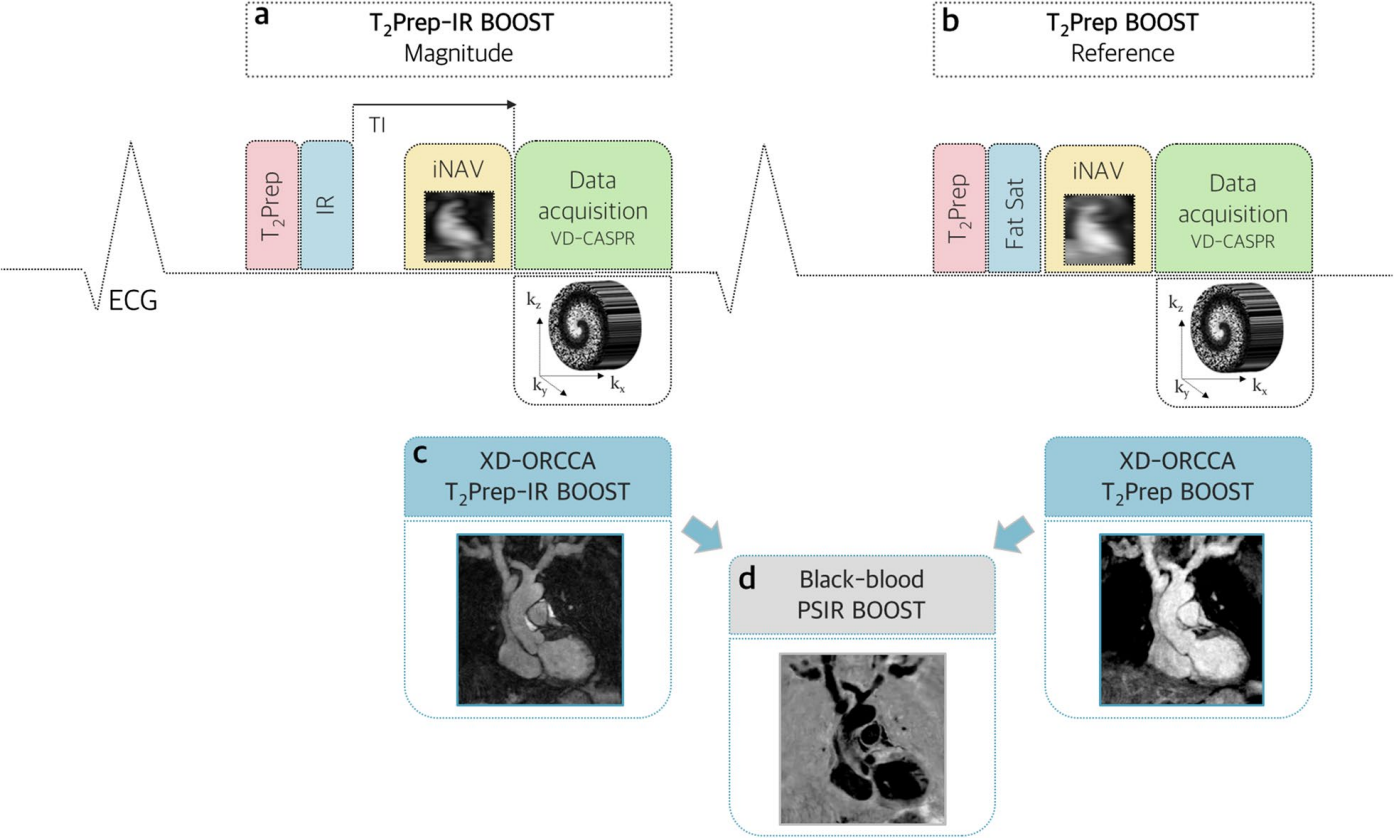
Proposed 3D whole-heart high-resolution motion-resolved accelerated T_2_-prepared Bright-blood and black-blOOd phase SensiTive inversion recovery (BOOST) for simultaneous black-blood late gadolinium enhancement (LGE) and bright-blood coronary cardiovascular magnetic resonance angiography (CCMRA). **a** A T_2_-prepared (T_2_Prep) inversion recovery (IR) module is applied at odd heartbeats (T_2_Prep-IR BOOST, magnitude image), whereas a **b** T_2_-prepared module is acquired at even heartbeats (T_2_Prep BOOST, reference image). In the latter, a SPIR pulse is used for fat saturation. In the former, the inversion time (TI) is set to null the signal from healthy viable myocardium and the fat signal is suppressed using a STIR-like fat approach. Both acquisitions are performed using a variable-density 3D Cartesian trajectory with spiral profile order and a high flip-angle of 90°. A low-resolution 2D image navigator (iNAV) is acquired at every heartbeat, before each spiral interleave of the 3D data acquisition, to estimate translational respiratory motion along the superior-inferior and left–right directions. **c** This motion information is used in the XD-ORCCA reconstruction to generate high-quality respiratory-resolved motion-compensated T_2_Prep-IR BOOST and T_2_Prep BOOST images from undersampled acquisitions. Then, the respiratory bin images are combined in a PSIR reconstruction to **d** generate a black-blood LGE image (PSIR BOOST) for scar visualization. The bright-blood T_2_Prep BOOST image (reference image, **b**) allows for coronary lumen visualization

The T_2_-prepared BOOST sequence alternates the acquisition of a T_2_-prepared IR module in odd heartbeats (T_2_Prep-IR, magnitude image) and a T_2_-prepared module in even heartbeats (T_2_Prep, reference image), as shown in Fig. 1a and b. Both undersampled datasets are acquired using a 3D variable-density Cartesian trajectory with spiral profile order (VD-CASPR) [23–25]. A short tau inversion recovery (STIR)-like fat suppression approach [26] is used in odd heartbeats, whereas a spectral pre-saturation (SPIR) [27] is used to suppress epicardial fat signal in even heartbeats. Low-resolution 2D iNAVs are acquired at every heartbeat to estimate 2D beat-to-beat translational motion (left–right: LR and superior-inferior: SI) and enable 100% respiratory scan efficiency and a predictable scan time. Motion estimation is performed independently for both BOOST datasets.

In post-contrast BOOST a subject-specific TI is set to null the signal from the healthy viable myocardium, whereas in non-contrast-enhanced BOOST a short TI = 110 ms is set to null the fat signal.

### XD-ORCCA reconstruction

**Fig. 2:**
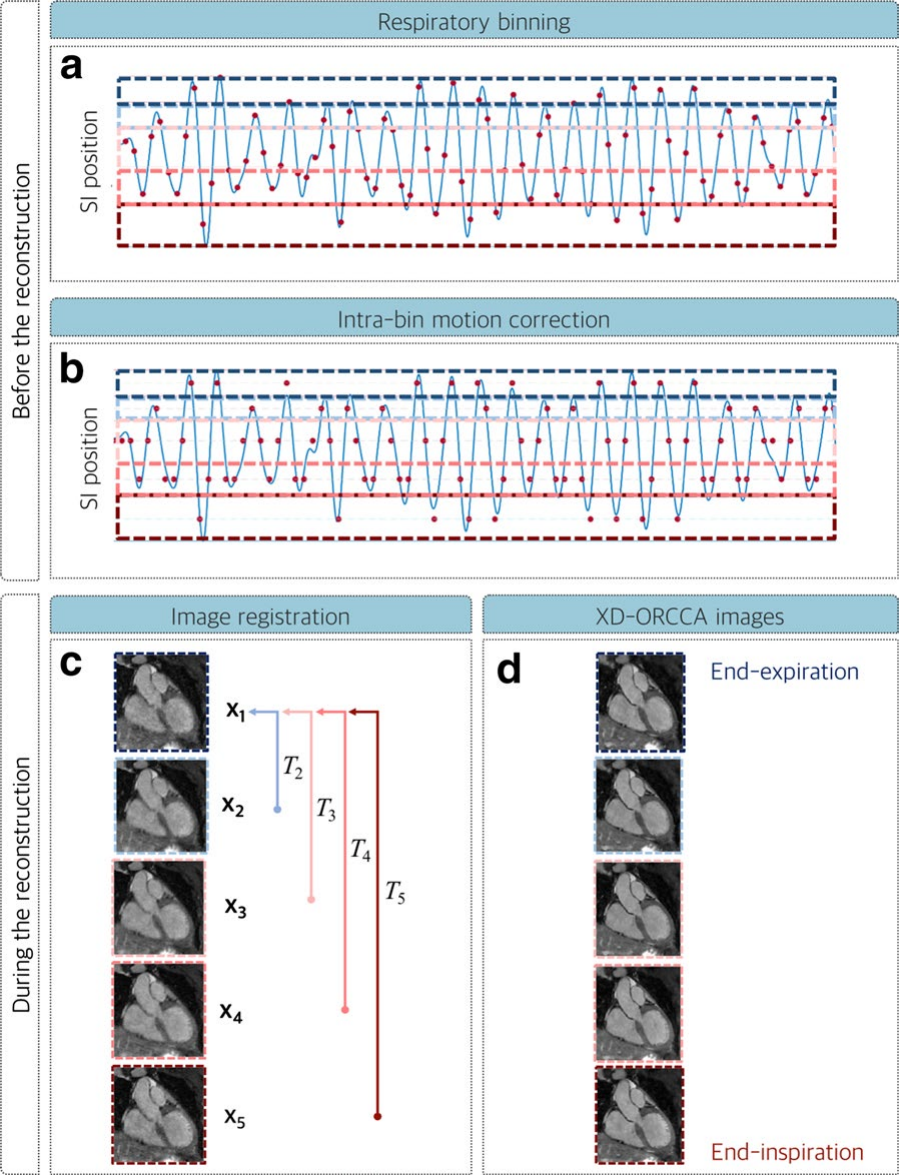
XD-ORCCA reconstruction: Low-resolution 2D iNAVs are acquired at every heartbeat and used to estimate beat-to-beat 2D translational respiratory motion along the superior-inferior (SI) and left–right (LR) directions. **a** The estimated SI motion information is used to separate the T_2_Prep-IR BOOST and T_2_Prep BOOST datasets into five different respiratory phases (or bins) each. **b** Moreover, 2D translational motion correction within each bin is performed in k-space before reconstruction to minimize intra-bin motion. In this step, data within each bin are corrected to the corresponding average bin position. **c** Finally, during image reconstruction, intra-bin motion-corrected images (x_b_) are aligned to a reference respiratory position (end-expiration) using the 2D translational motion information (T_b_), in order to increase the sparsity in the respiratory dimension and **d** generate high-quality motion-compensated respiratory-resolved images

The iNAVs are registered to a common respiratory position (end-expiration) and the 2D translational motion parameters are obtained via intensity-based image registration, using mutual information as a similarity measure and steepest gradient descent optimization. Then, the estimated SI information is used to obtain the respiratory signal, which is used to distribute each BOOST dataset into five equally populated respiratory bins (Fig. 2a). Subsequently, 2D translational motion correction within each bin is performed in k-space before respiratory-resolved image reconstruction to minimize intra-bin motion (Fig. 2b). This motion information is also used during the reconstruction to align the intra-bin motion-corrected images to end-expiration (Fig. 2c), in order to increase the sparsity in the respiratory domain [22]. Therefore, respiratory-resolved T_2_Prep-IR BOOST and bright-blood T_2_Prep BOOST images (Fig. 2d) are obtained by solving a modified version of XD-ORCCA, which explores the temporal sparsity in both forward and backward directions, instead of a unique direction, to further increase the sparsity [22]:

1 $${\hat{\mathbf{x}}} = {\text{arg }}\mathop {\text{min }}\limits_{{\mathbf{x}}} \left\{ { \frac{1}{2}{{\parallel\mathbf{Ex}} - {\mathbf{d}\parallel}}_{2}^{2} + \alpha \Psi_{{\text{t}}} \left( {{\mathcal{R}}{\mathbf{x}}} \right) + \beta \Psi_{{ - {\text{t}}}} \left( {{\mathcal{R}}{\mathbf{x}}} \right) + \gamma \Psi_{{\text{s}}} \left( {\mathbf{x}} \right)} \right\},$$

where **d** are the 2D intra-bin translational motion-corrected k-space data, ${\Psi }_{\mathrm{s}}$ is the 3D spatial total variation (TV) function, *α, β* and γ are regularization parameters, $\mathcal{R}\mathbf{x}={T}_{b}{\mathbf{x}}_{b}$ is the motion-corrected domain, where ${T}_{b}$ is the translation transform that maps the respiratory bin image ${\mathbf{x}}_{b}$ to the reference image ${\mathbf{x}}_{1}$ (end-expiration), ${\Psi }_{\mathrm{t}}={\Vert {{{T}_{b}\mathbf{x}}_{b}- T}_{b+1}{\mathbf{x}}_{b+1}\Vert }_{1}$ and ${\Psi }_{-t}={\Vert {{{T}_{b}\mathbf{x}}_{b}- T}_{b-1}{\mathbf{x}}_{b-1}\Vert }_{1}$ are 1D temporal TV functions in the forward and backward directions, respectively. The operator $\mathbf{E}={\mathbf{A}}_{{\varvec{b}}}\mathbf{F}\mathbf{S}$ incorporates the sampling matrix $\mathbf{A}$ for each bin *b*, Fourier transform $\mathbf{F}$ and coil sensitivities $\mathbf{S}$. The regularization parameters were selected empirically. Finally, the T_2_Prep-IR BOOST and bright-blood T_2_Prep BOOST images from the bin with the smallest respiratory SI displacement (usually end-expiration, Fig. 1c) are combined in a PSIR reconstruction to generate a 3D black-blood PSIR BOOST image (Fig. 1d).

### In-vivo experiments

All acquisitions reported in this study were performed on a 1.5T CMR system (MAGNETOM Aera, Siemens Healthineers, Erlangen, Germany) using an 18-channel chest-coil and a 32-channel spine coil. The study was approved by the National Research Ethics Service (1/11/12 and 15/NS/0030) and written informed consent was obtained from each participant according to institutional guidelines.

The 2D iNAVs were acquired using 14 ramp-up preparation pulses of the 3D whole-heart balanced steady state free precession (bSSFP) sequence with the same field-of-view (FOV) and orientation as the 3D BOOST acquisition.

#### Non-contrast-enhanced BOOST acquisitions: selection of undersampling factor

In-vivo free-breathing non-contrast-enhanced 3D BOOST acquisitions were performed in 12 healthy subjects (32 ± 8 years, 5 males, heart rate 57 beats/minute). Fully-sampled BOOST (with elliptical k-space coverage), 2.6 × and 3.8 × accelerated acquisitions were performed in a randomized order for each subject using the following parameters: coronal orientation, FOV = 320 × 320 × 96–112 mm^3^, resolution = 1 × 1 × 2 mm^3^, (repetition time) TR/ (echo time) TE = 3.6/1.56 ms, pixel bandwidth = 977 Hz/pixel, flip angle = 90°, TI = 110 ms, T_2_Prep = 40 ms, subject-specific mid-diastolic trigger delay and acquisition window (corresponding to 28–40 k-space readouts per heartbeat). Acquisition time for the fully-sampled, 2.6 × and 3.8 × accelerated non-contrast-enhanced BOOST approach was 17:12 ± 2:5 min, 8:38 ± 1:3 min, and 5:36 ± 0:46 min, respectively.

The bright-blood T_2_Prep BOOST images (interpolated to 0.6 × 0.6 × 0.6 mm^3^) were reformatted using SoapBubble [28] to allow simultaneous visualization of the right coronary artery (RCA), left anterior descending coronary artery (LAD) and whenever possible the left circumflex coronary artery (LCX). Using the same software, coronary percentage vessel sharpness (first 4 cm and full length) and visible vessel length of both RCA and LAD were measured for all acquisitions. A Two One-Sided Test of Equivalence (TOST), as described in [29], was used to compare the quantitative results obtained from undersampled acquisitions to those obtained from the fully-sampled reference. If p-value < 0.05, the test rejects the null hypothesis of non-equivalence and accepts the alternative hypothesis of equivalence.

#### Contrast-enhanced BOOST acquisitions

Free-breathing contrast-enhanced 3D whole-heart BOOST and conventional breath-hold 2D PSIR LGE acquisitions were performed in 10 patients (66 ± 15 year, 7 males, average heart rate 58 beats/minute) with suspected cardiovascular disease. First, the multi-slice and multi-breath-hold 2D PSIR LGE clinical sequence (four chamber view; three chamber view; short-axis view) was acquired ~ 10 min after Gadobutrol (Gadovist, Bayer Healthcare, Berlin, Germany) injection (0.2 mmol/kg) with bSSFP readout, in-plane spatial resolution = 1.4 × 1.4 mm^2^, slice thickness = 8 mm, 10–12 slices for the short axis view, FOV = 340 × 360 mm^2^, TR/TE = 2.9/1.2 ms, pixel bandwidth = 780 Hz/pixel, flip angle = 45°, and subject-specific mid-diastolic trigger delay. The TI (ranging in the interval 225–310 ms) was selected with a dedicated TI scout scan and was set to null the signal from the healthy myocardium.

At the end of the clinical protocol, post-contrast BOOST acquisitions were performed (~ 25–40 min after injection) using the following parameters: coronal orientation, FOV = 320 × 320 × 96–128 mm^3^, resolution = 1 × 1 × 2 mm^3^, TR/TE = 3.6/1.57 ms, pixel bandwidth 977 Hz/pixel, flip-angle = 90°, T_2_Prep = 40 ms, 3.8-fold undersampling, subject-specific mid-diastolic trigger delay (corresponding to 20–40 k-space readouts per heartbeat) and acquisition time 7:14 ± 2:57 min. The subject-specific TI (90–150 ms) was selected to null the signal from viable healthy myocardium and was identified using a dedicated 2D BOOST TI scout scan during breath-hold. The 2D BOOST TI scout scan consisted of a magnetization-prepared cine sequence with a T2Prep-IR module applied right after the R-wave at odd heartbeats and T2-preparation only at even heartbeats [30].

Blinded qualitative grading of anonymized clinical 2D PSIR and 3D PSIR BOOST images was performed on consensus basis by three cardiologists (T.F.I, I.R., SCMR III accredited, and R.H., SCMR II accredited). Images were stored as grayscale digital imaging and communications in medicine (DICOM) images and were windowed so that the black-blood PSIR BOOST showed a black blood pool, which was darker than the healthy myocardium, whereas in the clinical 2D PSIR the normal myocardium was black (with noise still visible). LGE uptake was bright in both 3D PSIR BOOST and 2D PSIR. The diagnostic quality of the clinical 2D PSIR and 3D PSIR BOOST images was graded using a 4-point scale: (1) non-diagnostic, (2) diagnostic with major artifacts, (3) diagnostic with minor artifacts and (4) fully diagnostic with no artifacts. LGE visualization scores were compared using a nonparametric TOST based on the Wilcoxon signed-rank test [31]. The level of significance of the p-value was 0.05. The diagnostic quality of the proximal, mid and distal sections of the LAD, LCX, and RCA course, was graded using the same 4-point scale. BOOST images whose acquisition started later than 40 min after contrast injection were not considered in the analysis, because the relative contrast between normal and diseased myocardium significantly decreases due to contrast agent washout.

## Results

### Non-contrast-enhanced BOOST acquisitions: selection of undersampling factor

**Fig. 3:**
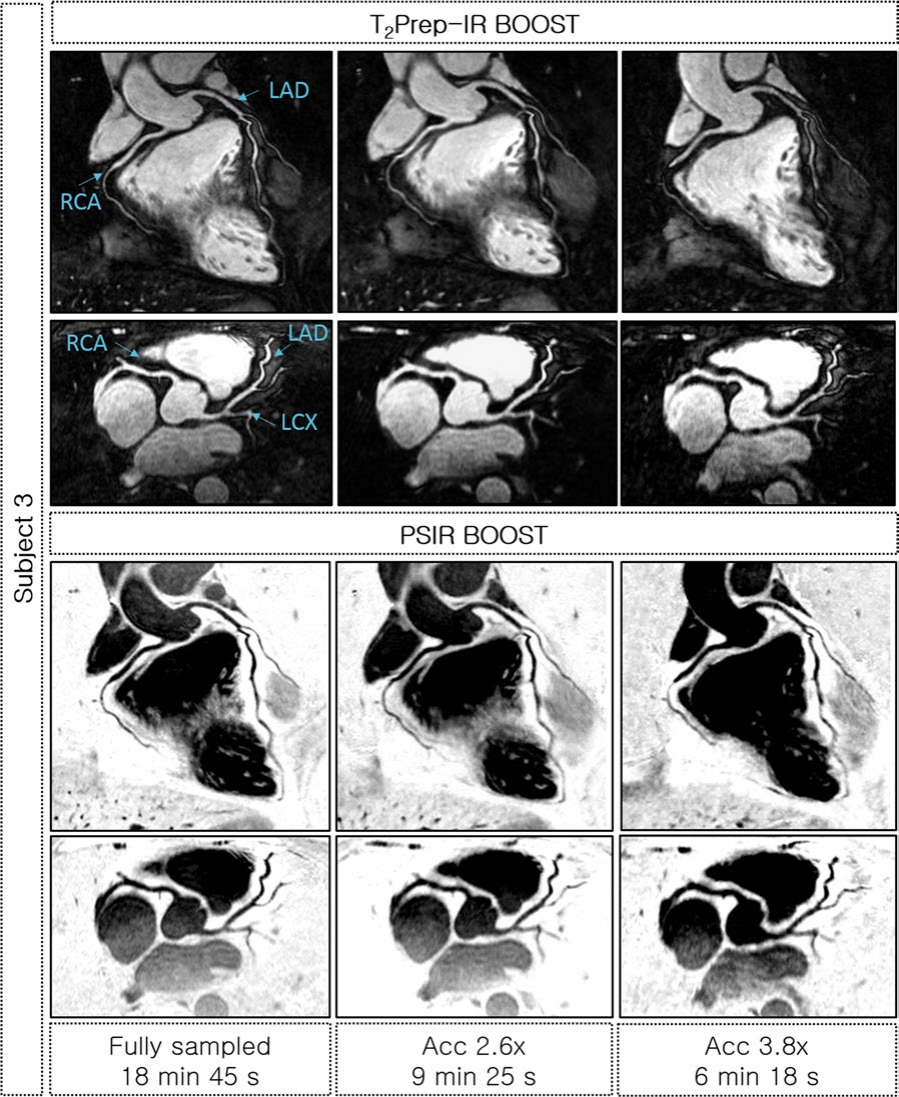
Reformatted T_2_Prep-IR BOOST and PSIR BOOST images obtained for one representative healthy subject using the proposed framework. Images were reconstructed from fully-sampled, 2.6 × and 3.8 × undersampled BOOST datasets and display the right (RCA), left anterior descending (LAD) and left circumflex artery (LCX) coronary arteries. Images obtained from accelerated BOOST acquisitions have similar quality to those obtained from the fully-sampled BOOST acquisition, allowing for clear visualization of the coronary arteries. Total acquisition times are indicated for each acquisition

**Fig. 4:**
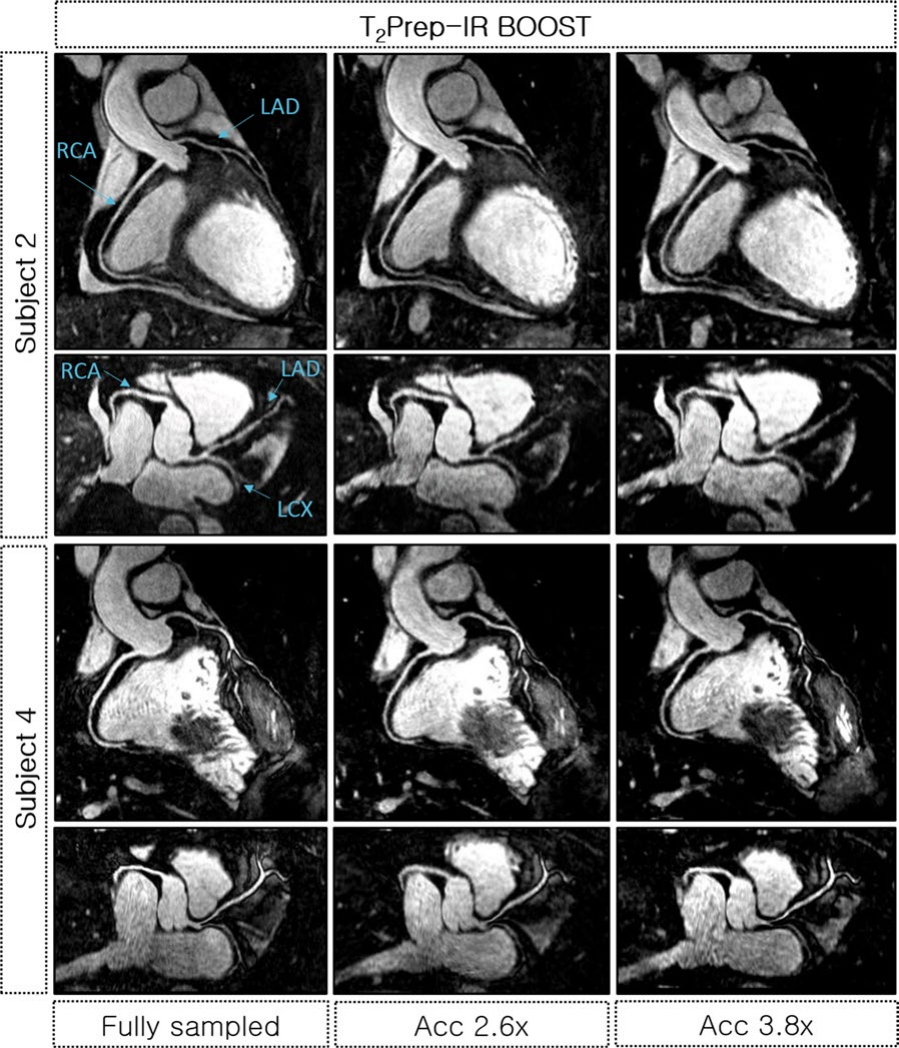
Reformatted T_2_Prep-IR BOOST images obtained for two representative healthy subjects, displaying the right (RCA), left anterior descending (LAD) and left circumflex (LCX) coronary arteries. Images were reconstructed from fully-sampled, 2.6 × and 3.8 undersampled BOOST datasets. Images obtained from accelerated acquisitions have comparable quality to those obtained from fully-sampled BOOST acquisitions

Free-breathing BOOST acquisitions and motion-resolved reconstructions with intra-bin motion compensation were successfully obtained for all 12 subjects. The reformatted bright-blood T_2_Prep BOOST and black-blood PSIR BOOST images (respiratory phase with the smallest SI respiratory displacement) obtained using the proposed method from fully-sampled, 2.6 × and 3.8 × undersampled acquisitions for a representative healthy subject are shown in Fig. 3. In addition, the reformatted bright-blood T_2_Prep BOOST images obtained from fully-sampled and undersampled data for another two representative healthy subjects are shown in Fig. 4. For subject 3 (Fig. 3, right), the tortuous anatomy of the RCA prevented an appropriate reformatting of the mid segment of the vessel. However, the mid-RCA was successfully reconstructed from accelerated BOOST acquisitions using the proposed method (Additional file 1).

**Fig. 5:**
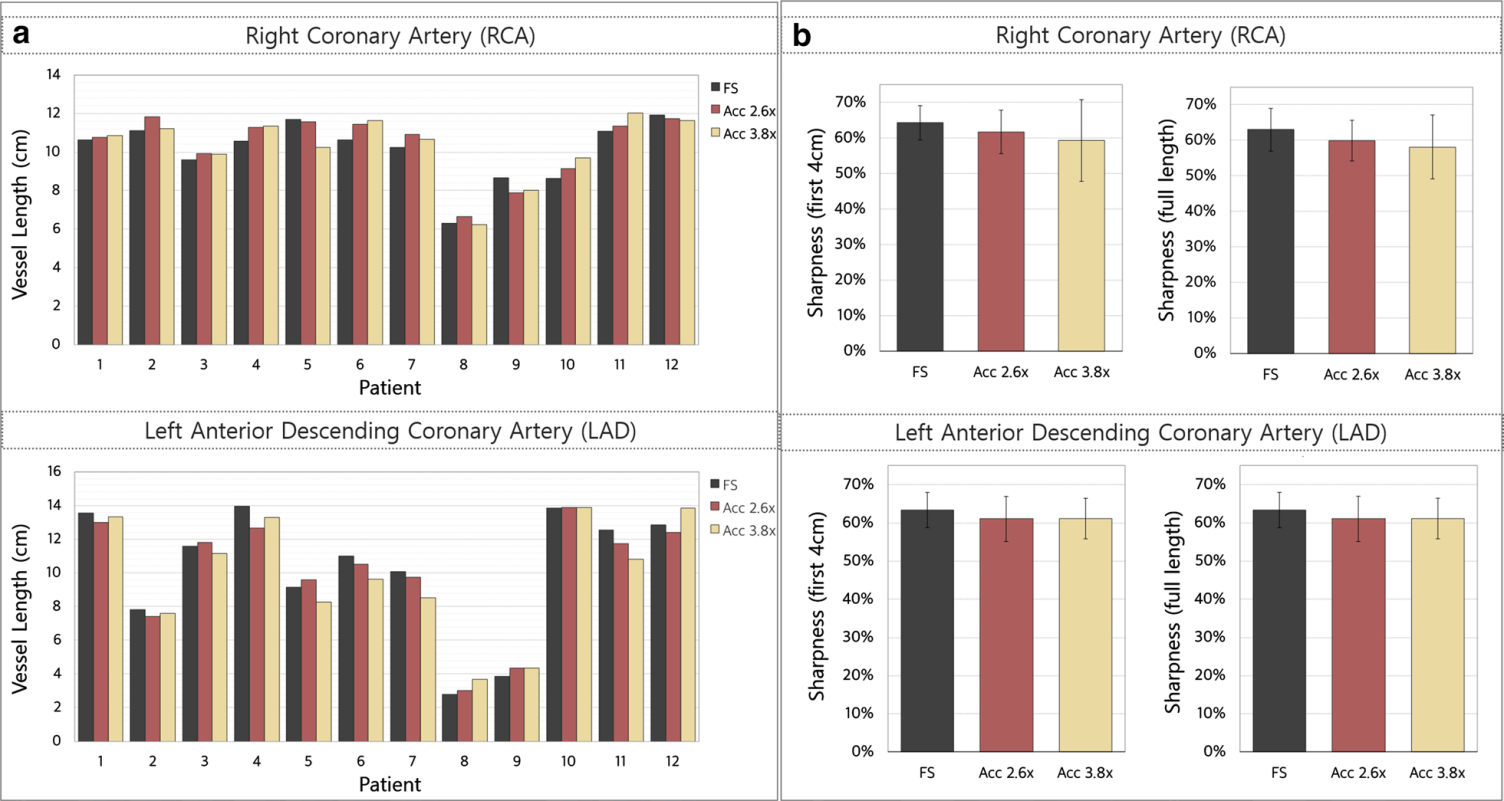
**a** Vessel length of the right (RCA) and left anterior descending (LAD) coronary arteries for all 12 healthy subjects obtained from the T_2_Prep-IR BOOST reconstructions of fully-sampled (FS), 2.6 × and 3.8 × accelerated (Acc) BOOST datasets. Significant equivalence (p < 0.05) in visible vessel length was observed between the reconstructions obtained from fully-sampled and accelerated acquisitions, for both coronary arteries. **b** Vessel sharpness obtained from the fully-sampled and accelerated T_2_Prep-IR BOOST images for all 12 healthy subjects. Image quality was assessed by measuring the first 4 cm and full length of the RCA and LAD. Measurements of vessel sharpness obtained for both coronary arteries were statistically equivalent (p < 0.05)

The RCA and LAD visible vessel lengths measured from the images reconstructed from fully-sampled, 2.6 × and 3.8 × undersampled datasets are shown in Fig. 5a. The average visible vessel lengths for the RCA were 10.1 ± 1.6 cm, 10.4 ± 1.7 cm and 10.3 ± 1.7 cm for the fully-sampled, 2.6 × and 3.8 × undersampled datasets, respectively. For the LAD, the corresponding average vessel lengths were 10.3 ± 3.8 cm, 10.0 ± 3.5 cm and 9.9 ± 3.5 cm. Statistically significant equivalence (p < 0.05) was found in visible vessel length when comparing measurements from fully-sampled and undersampled BOOST images, for both coronary arteries. Additionally, statistically significant equivalence was found in the sharpness of both coronary arteries between the images obtained from fully-sampled and undersampled BOOST acquisitions (Fig. 5b). The vessel sharpness for the first 4 cm of the RCA was (fully-sampled) 64 ± 5%, (2.6 ×) 62 ± 6% and (3.8x) 59 ± 11%. For the LAD the vessel sharpness was (fully-sampled) 63 ± 5%, (2.6x) 61 ± 6% and (3.8 ×) 61 ± 5%.

The proposed method produces images from 2.6 × and 3.8 × accelerated BOOST acquisitions with comparable visual quality to those obtained from fully-sampled BOOST acquisitions (Additional file 2).

### Contrast-enhanced BOOST acquisitions

Clinical and demographic characteristics are summarized in Table 1. Patient 6 and patient 9 were not included in the qualitative analysis because BOOST data acquisition started more than 40 min after contrast agent injection. In addition, for patient 6, residual motion artifacts were visible in the BOOST images, which resulted from the rigid translation of the chest walls and arms. Moreover, patient 9 exhibited very irregular heartbeats, very short diastolic window and was not able to breath-hold, making the acquisition of CMR exam images very challenging, including the clinical 2D PSIR images, which further delayed the BOOST acquisition. Therefore, image quality analysis was performed for 8 of 10 patients. The average time after injection for BOOST acquisitions was 32 ± 7 min.

**Table 1:** Summary of patients’ data. In addition, the presence of LGE findings in the clinical 2D PSIR images is stated. Patients 6 and 9 were not considered for the quantitative analysis since BOOST acquisitions started later than 40 min after contrast injection

Patient	Gender	Age (years)	Weight (kg)	Height (m)	BMI (kg/m^2^)	HR(bpm)	Clinical condition	Positive LGE
1	M	79	76	1.62	29	56	Dilated cardiomyopathy and myocardial infarction	Yes
2	M	68	66	1.47	31	52	Severe LV impairment and myocardial infarction	Yes
3	F	48	64	1.65	24	54	Dilated cardiomyopathy and fibrosis	No
4	M	75	71	1.63	27	48	Dilated cardiomyopathy	No
5	F	72	66	1.63	25	48	Myocarditis	No
6	F	49	82	1.63	31	60	Myocardial infarction	Yes
7	M	44	91	1.79	28	66	Myocarditis	Yes
8	M	84	60	1.75	20	54	Ischemic cardiomyopathy	No
9	M	82	101	1.81	31	70	Myocardial infarction	No
10	M	55	67	1.65	25	86	Normal	No

*BMI* body mass index, *HR* heart rate, *bpm* beats per minute, *LV* left ventricle

All eight 3D PSIR BOOST datasets were considered diagnostic, with a median grade of 4 (range 3–4); specifically, 5/8 cases were graded 4 and 3 cases were graded 3. Clinical 2D PSIR images were graded 4 for 4 patients, 3 cases were graded 3 and one case was graded 2 (median score 3.5, range 2–4). In terms of visual grading, the 3D PSIR BOOST datasets and the clinical 2D PSIR counterpart were found to be statistically equivalent (p < 0.05).

**Fig. 6:**
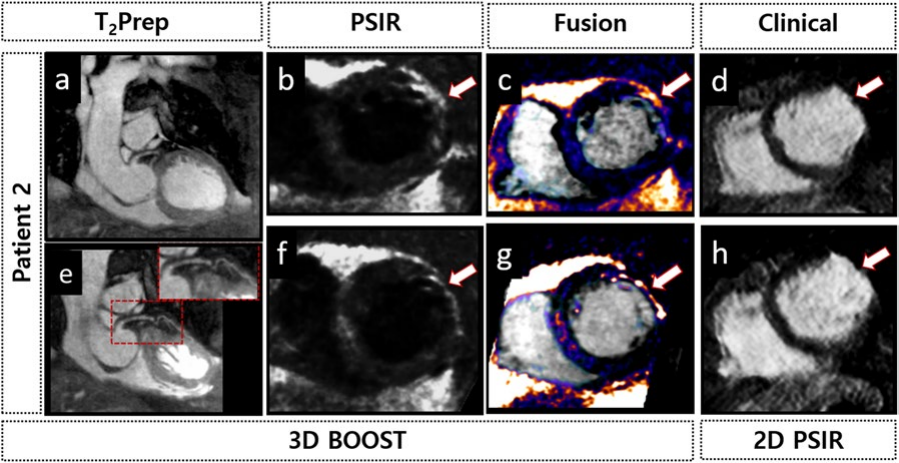
Accelerated high-resolution 3D BOOST and clinical 2D PSIR images for one representative patient with myocardial infarction. **a** Bright-blood T_2_Prep BOOST image (coronal view) and **e** corresponding reformatted image showing the LAD. **b**, **f** BOOST provides a black-blood PSIR image for LGE scar visualization (arrows), which shows good agreement with (**d**, **h**) clinical 2D PSIR. **c**, **g** Fusion between the bright-blood T_2_Prep BOOST and the black-blood PSIR BOOST images allows the assessment of scar location and transmurality. The signal from both blood and viable myocardium is nulled in black-blood PSIR BOOST images, thus providing images with good contrast between the blood pool and scar tissue. LGE uptake can be seen (arrows) in the short-axis views **b**, **c**, **d**, **f**, **g**, **h** of the mid (top) and basal (bottom) anterolateral walls. Clinical 2D PSIR and BOOST were acquired ~ 10 min and ~ 35 min post-contrast, respectively

**Fig. 7:**
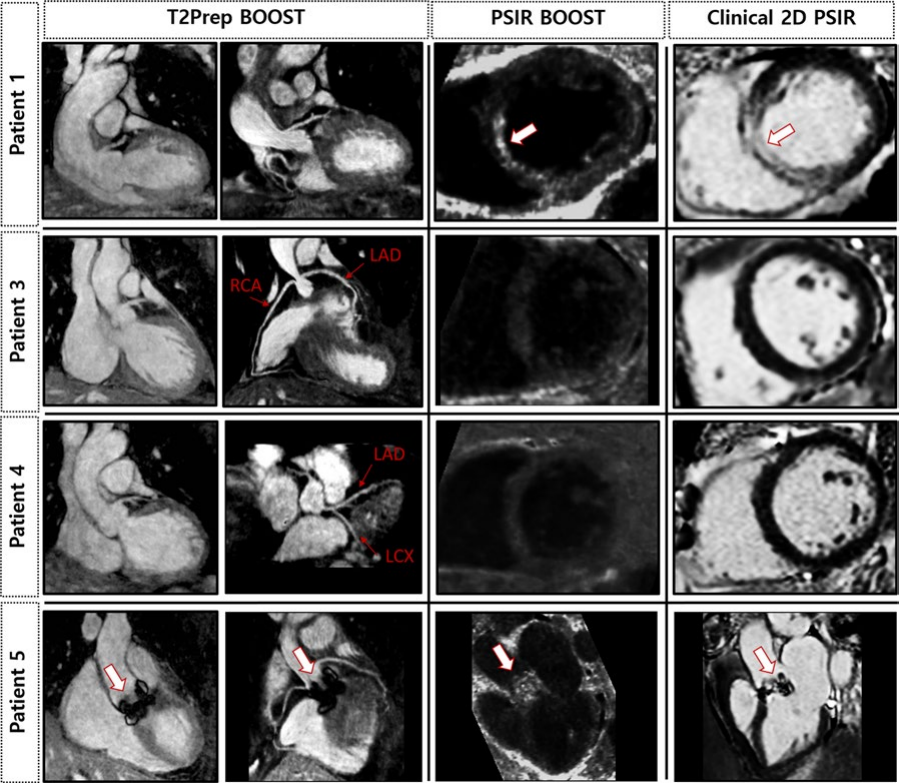
Accelerated high-resolution 3D BOOST and clinical 2D PSIR images obtained for four patients. (left) Bright-blood T_2_Prep BOOST coronal views for visualization of heart anatomy are displayed together with coronary reformats showing the left anterior descending (LAD), left circumflex (LCX), and right (RCA) coronary arteries. (middle) 3D black-blood PSIR BOOST images and (right) clinical bright-blood 2D PSIR images. Patient 1 had myocardial infarction and septal LGE uptake is visible in both PSIR BOOST and clinical 2D PSIR short-axis views (arrows). Patients 3, 4 and 5 had non-ischaemic cardiomyopathyies with no LGE uptake. Moreover, Patient 5 had a bioprosthetic aortic valve replacement, which is nicely depicted in the bright-blood T_2_Prep image (arrows)

The clinical 2D PSIR and 3D BOOST images (T_2_Prep, PSIR and fusion of T_2_Prep and PSIR images) are shown in Fig. 6 for a patient with myocardial infarction. LGE uptake can be seen in the mid and basal anterolateral walls in both 3D PSIR BOOST and 2D clinical images. Some LGE uptake can also be seen in the septal wall in the 3D PSIR BOOST. Moreover, bright‐blood T_2_Prep and black‐blood PSIR BOOST images are shown in Fig. 7 for four representative patients, together with the clinical 2D PSIR acquisitions.

**Fig. 8:**
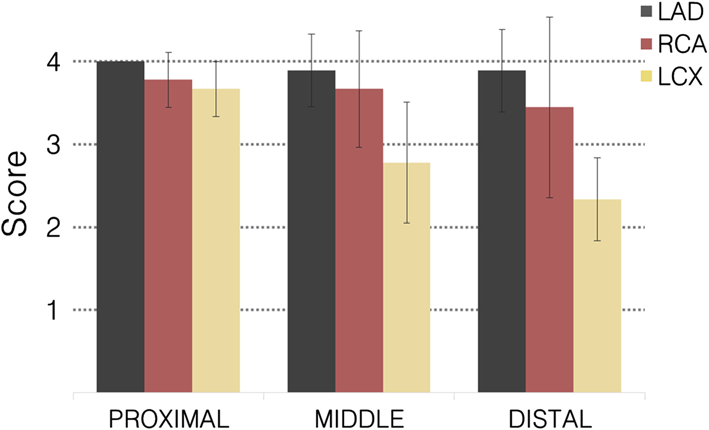
Qualitative visual scores obtained for all eight patients for the proximal, middle and distal sections of the left anterior descending (LAD), right (RCA) and left circumflex (LCX) coronary arteries. Depiction of coronary artery course was graded using a 4-point scale system (1: non-diagnostic, 2: diagnostic with major artifacts, 3: diagnostic with minor artifacts and 4: fully diagnostic with no artifacts). Bright-blood T_2_Prep BOOST images allowed for visualization of the proximal and middle LAD, LCX and RCA segments with high diagnostic quality. All segments had diagnostic value

The LGE findings present in the 2D clinical PSIR images of patient 7 were reported as a non-ischemic cardiomyopathy (myocarditis). However, in the 3D BOOST PSIR images, LGE uptake was identified as a transmural myocardial infarction. Therefore, correspondence between clinical 2D PSIR and 3D PSIR BOOST images was found in all the cases except for one patient with a non-ischemic cardiomyopathy. In addition, the consensus grading scores indicate that the bright-blood T_2_Prep BOOST images allowed for visualization of the proximal and middle LAD, LCX and RCA sections with high diagnostic quality (Fig. 8) and visualization of the distal sections of the RCA and LCX was fair. Nevertheless, all segments ultimately had diagnostic value.

## Discussion

In this work, the respiratory-resolved motion-corrected XD-ORCCA reconstruction has been extended to accelerate the T_2_-prepared BOOST sequence and achieve high-resolution motion-corrected 3D whole-heart black-blood LGE and CCMRA, within clinically feasible acquisition times. Respiratory-resolved images are obtained for each BOOST dataset (T_2_Prep-IR and T_2_Prep) using XD-ORCCA, which uses 2D translational motion information extracted from iNAVs to increase the sparsity in the respiratory dimension and to compensate for residual motion within each respiratory bin. Then, the T_2_Prep-IR and T_2_Prep BOOST images are combined in a PSIR reconstruction to generate a dark-blood PSIR BOOST image.

Initially, the feasibility of using XD-ORCCA to accelerate the T_2_-prepared BOOST acquisitions was tested using non-contrast-enhanced 3D BOOST acquisitions in healthy subjects. Fully-sampled BOOST acquisitions were compared against 2.6-fold and 3.8-fold accelerated BOOST scans. This study showed that the proposed framework produces high quality motion-compensated images from 2.6-fold and 3.8-fold accelerated free-breathing BOOST acquisitions. Equivalence tests with TOST revealed statistically significant equivalence in visible vessel length and sharpness when comparing measurements from fully-sampled bright-blood BOOST image and undersampled BOOST images, for both coronaries. Therefore, acquisition times can be reduced from ~ 17 to ~ 6 min by using 3.8-fold accelerated BOOST acquisitions, without significantly sacrificing image quality. However, the fixed TI value used in this study is not the optimal for all subjects since TI is sensitive to the heart rate. Therefore, image quality could be improved by using of a subject-specific TI to suppress the fat signal and reduce chemical shift artefacts, such as those observed in Fig. 3.

The first study, using non-contrast-enhanced 3D BOOST acquisitions, was essential to select the optimal acceleration factor to be used in the contrast-enhanced BOOST study. It showed that XD-ORCCA can reconstruct images from 3.8 × accelerated BOOST acquisitions with comparable quality to those obtained from fully-sampled acquisitions. Thus, in the second study, 3.8-fold accelerated post-contrast BOOST acquisitions were compared against clinical 2D PSIR in patients with suspected coronary artery disease. All 3D BOOST PSIR images included in the qualitative study had diagnostic quality. Moreover, scar tissue could be identified in 3D PSIR BOOST images despite image acquisition only starting on average 32 min after contrast administration. This was facilitated by the high contrast between scar tissue and blood pool in 3D PSIR BOOST images compared to clinical 2D PSIR. Healthy viable myocardium and scar tissue (when present) were visible in matching anatomical locations for both the clinical 2D PSIR and the 3D whole-heart PSIR BOOST images. In addition, bright-blood T_2_Prep BOOST images allowed visualization of the origin, proximal and mid sections of the coronary arteries with high diagnostic quality. Hence, the proposed framework successfully generates high-resolution 3D whole-heart black-blood PSIR LGE and bright-blood coronary angiography images with sufficient diagnostic quality from 3.8-fold accelerated post-contrast BOOST acquisitions, which can be obtained in a clinically feasible scan time (~ 7 min).

For patient 2, septal LGE uptake was identified in the 3D BOOST PSIR, but was not detected in the 2D clinical PSIR (Fig. 6). This finding could be because the proposed method provides high-resolution (1 × 1 × 2 mm^3^) black-blood PSIR BOOST images with whole-heart volumetric coverage, whereas the clinical 2D PSIR images have lower in-plane resolution (1.4 × 1.4 mm^2^) and large slice thickness (8 mm). Moreover, clinical 2D PSIR requires multiple breath-holds, and hence, 2D PSIR images may not be in the same respiratory phase as the displayed 3D PSIR BOOST images. However, to draw conclusions, further studies in a large cohort of patients are necessary to compare the location and extent of LGE in accelerated 3D PSIR BOOST and clinical 2D PSIR.

The number of respiratory bins used in both studies provides a good compromise between remaining intra-bin motion and undersampling artifacts [32]. However, better results could potentially be achieved by adapting the number of respiratory bins according to the irregularity and amplitude of the respiratory motion. One solution would be to add/remove respiratory bins until a fixed bin width is achieved (e.g. 3 mm). Additionally, arrhythmia rejection techniques can be integrated with the proposed framework to further improve imaging quality. At present, the sequence requires relatively regular R-R intervals and therefore cannot be used in patients with atrial fibrillation, although this is a limitation for many techniques employed in clinical CMR.

Currently, it is assumed that the T_2_Prep-IR BOOST and T_2_Prep BOOST images generated with XD-ORCCA are registered, and hence, in the same respiratory position. However, there could potentially be registration errors, which can cause phase errors in the PSIR BOOST images. The use of non-rigid registration to correct for residual non-rigid motion between the highest-quality T_2_Prep-IR BOOST and bright-blood T_2_Prep BOOST bin images was tested in a preliminary study in healthy subjects, showing that the visual quality of the final 3D black-blood PSIR BOOST image could be slightly improved (Additional file 3).

The proposed framework only considers translation motion along the SI and LR direction. However, respiratory-induced heart motion involves more complex motion patterns, including motion along the anterior–posterior direction and even non-rigid deformation. Therefore, residual motion could be addressed by estimating/correcting for 3D translational or non-rigid motion at each XD-ORCCA iteration. Alternatively, the high-quality respiratory XD-ORCCA images could be used to estimate 3D bin-to-bin non-rigid motion, which can subsequently be used to generate 3D T_2_Prep-IR BOOST and T_2_Prep BOOST non-rigid motion-corrected images [24]. Moreover, non-rigid respiratory motion correction may help reduce ghosting artefacts that may originate from rigid translation of static tissues such as the chest wall and arms. However, all these methods are computationally more expensive.

The proposed 3D PSIR BOOST requires separate reconstructions for each T_2_Prep-IR and T_2_Prep BOOST dataset. However, since this produces images of the same anatomical region with different contrasts, a joint sparsity regularization term could be added to Eq. (1) to exploit the structural similarity (sparsity) across contrasts and jointly reconstruct T_2_Prep-IR and T_2_Prep BOOST images. This approach could potentially provide higher quality images than reconstructing the T_2_Prep-IR and T_2_Prep BOOST images separately [33–35].

In this study, post-contrast BOOST acquisitions were performed at the end of the clinical CMR examination so that the research protocol did not interfere with the clinical CMR exam indicated to diagnose suspected cardiovascular disease. Therefore, injection of contrast agent was optimized to provide optimal contrast for the clinical 2D PSIR sequences and the order of the 2D PSIR and 3D BOOST acquisitions was not randomized as per ethical requirements. Thus, post-contrast BOOST acquisitions had potentially sub-optimal contrast conditions. This was particularly evident for two specific cases that were excluded of the qualitative analysis (patient 6 and patient 9). Moreover, additional challenges can occur by performing BOOST acquisitions at the end of the clinical protocol, for example, patients’ tolerance to being inside the scanner reduces with time and there can be more respiratory and heart rate variability that can have a detrimental effect on image quality. Therefore, accelerated high-resolution post-contrast 3D BOOST and clinical 2D PSIR acquisitions will be compared in a future study by randomizing the order of the two acquisitions and by performing separate contrast injections to ensure similar contrast conditions. Future studies will also aim to investigate the accuracy of black-blood PSIR BOOST for the detection and quantification of scar transmurality in patients with angiographically confirmed coronary artery disease.

Patient 7 had a non-ischemic cardiomyopathy (myocarditis), which was identified as a myocardial infarct in the BOOST PSIR images. This could be due the lack of contrast between the healthy myocardium and blood pool, which gives the impression that the fibrosis is sub-endocardial when, in fact, it is located in the mid myocardial wall. Moreover, BOOST imaging started more than 30 min post-contrast injection, and hence, contrast agent washout could have prevented adequate scar depiction. However, a more accurate location and delineation of scar tissue can be obtained by fusing the black-blood PSIR image with the bright-blood T_2_Prep BOOST image, which then allows both scar and myocardial anatomy visualization as shown in Fig. 6. Alternatively, a grey-blood PSIR technique can be used, which is optimized to null the blood pool, leading to a gray appearance of the blood in the PSIR image, and hence, offering better contrast between myocardium and blood pool [36, 37]. Nevertheless, the ability of the proposed method to discriminate between ischemic and non-ischemic cardiomyopathies needs to be evaluated and will also be the subject of future studies.

The accelerated high-resolution non-contrast-enhanced BOOST showed promising results for both bright blood and black‐blood coronary artery imaging. However, the suitability of the proposed method for detecting coronary thrombus still needs to be investigated. Nevertheless, this indicates that the proposed framework is quite versatile and could potentially be used with other free-breathing LGE or PSIR techniques [38, 39].

## Conclusions

An accelerated 3D whole-heart T_2_-prepared BOOST framework was proposed to provide high-resolution 3D whole-heart bright-blood and black-blood PSIR BOOST images within a clinically feasible scan time of ~ 7 min. This was achieved by combining the XD-ORCCA respiratory-resolved reconstruction method with free-breathing 3D whole-heart Cartesian BOOST undersampled acquisitions. The proposed method was tested in a group of cardiac patients and showed a good agreement with the clinical 2D PSIR LGE.

## Supplementary information

**Additional file 1: Fig. S1.** Subject 3 reconstructions (coronal views) obtained from fully-sampled, 2.6x and 3.8x accelerated T2Prep-IR BOOST data. The tortuous anatomy of the RCA prevented an appropriate multiplanar reformatting of the mid segment of the vessel. However, the zoomed areas (yellow boxes) of the non-reformatted coronal views show that the mid-RCA was successfully reconstructed from fully-sampled and accelerated BOOST acquisitions using the proposed method. Coronal view numbers are indicated in each subfigure.

**Additional file 2: Fig. S2.** Coronal views showing the T2Prep-IR BOOST and PSIR BOOST images reconstructed from fully-sampled, 2.6 x and 3.8x undersampled non-contrast-enhanced BOOST datasets, for a representative healthy subject. Images obtained from accelerated acquisitions have comparable quality to those obtained from fully-sampled acquisitions. The complete heart anatomy and coronary arteries can be clearly visualized in all the images.

**Additional file 3: Fig. S3.** 3D black-blood PSIR BOOST coronal views obtained (1st and 3rd rows) without (No MA) and (2nd and 4th rows) with non-rigid motion alignment (NMC) between the T2Prep-IR BOOST and T2Prep BOOST bin images before the PSIR reconstruction, for two representative healthy subjects. The proposed method assumes that the highest quality T2Prep-IR BOOST and T2Prep BOOST bin images are registered. However, there could be some residual non-rigid motion. Hence, non-rigid registration was used to correct for residual non-rigid motion between the T2Prep-IR BOOST and bright-blood T2Prep BOOST bin images. The visual quality of the final 3D black-blood PSIR BOOST image improved slightly when non-rigid motion alignment was used between the T2Prep-IR and T2Prep BOOST images.

## Data Availability

The datasets used and/or analysed during the current study are available from the corresponding author on reasonable request.

## Abbreviations

BOOST: Bright-blood and black-blOOd phase SensiTive inversion recovery
bSSFP: Balanced steady state free precession
CMR: Cardiovascular magnetic resonance
CCMRA: Coronary cardiovascular magnetic resonance angiography
FOV: Field of view
Gd: Gadolinium
iNAV: Image-based NAVigator
IR: Inversion recovery
LAD: Left anterior descending coronary artery
LCX: Left circumflex coronary artery
LGE: Late gadolinium enhancement
LR: Left–right
PSIR: Phase-sensitive inversion recovery
RCA: Right coronary artery
SI: Superior–inferior
STIR: Short tau inversion recovery
T_2_-Prep: T_2_-prepared
T_2_-Prep-IR: T_2_-prepared inversion recovery
TE: Echo time
TI: Inversion time
TOST: Two one-sided test of equivalence
TR: Repetition time
VD-CASPR: Variable density spiral like Cartesian trajectory
XD-ORCCA: Optimized respiratory‐resolved Cartesian coronary MR angiography
