# Supplementary figures and images for: Accelerated high-resolution free-breathing 3D whole-heart T2-prepared black-blood and bright-blood cardiovascular magnetic resonance
Source: J Cardiovasc Magn Reson. 2020 Dec 14;22:88. doi: 10.1186/s12968-020-00691-3 (PMC7737390; doi:10.1186/s12968-020-00691-3)

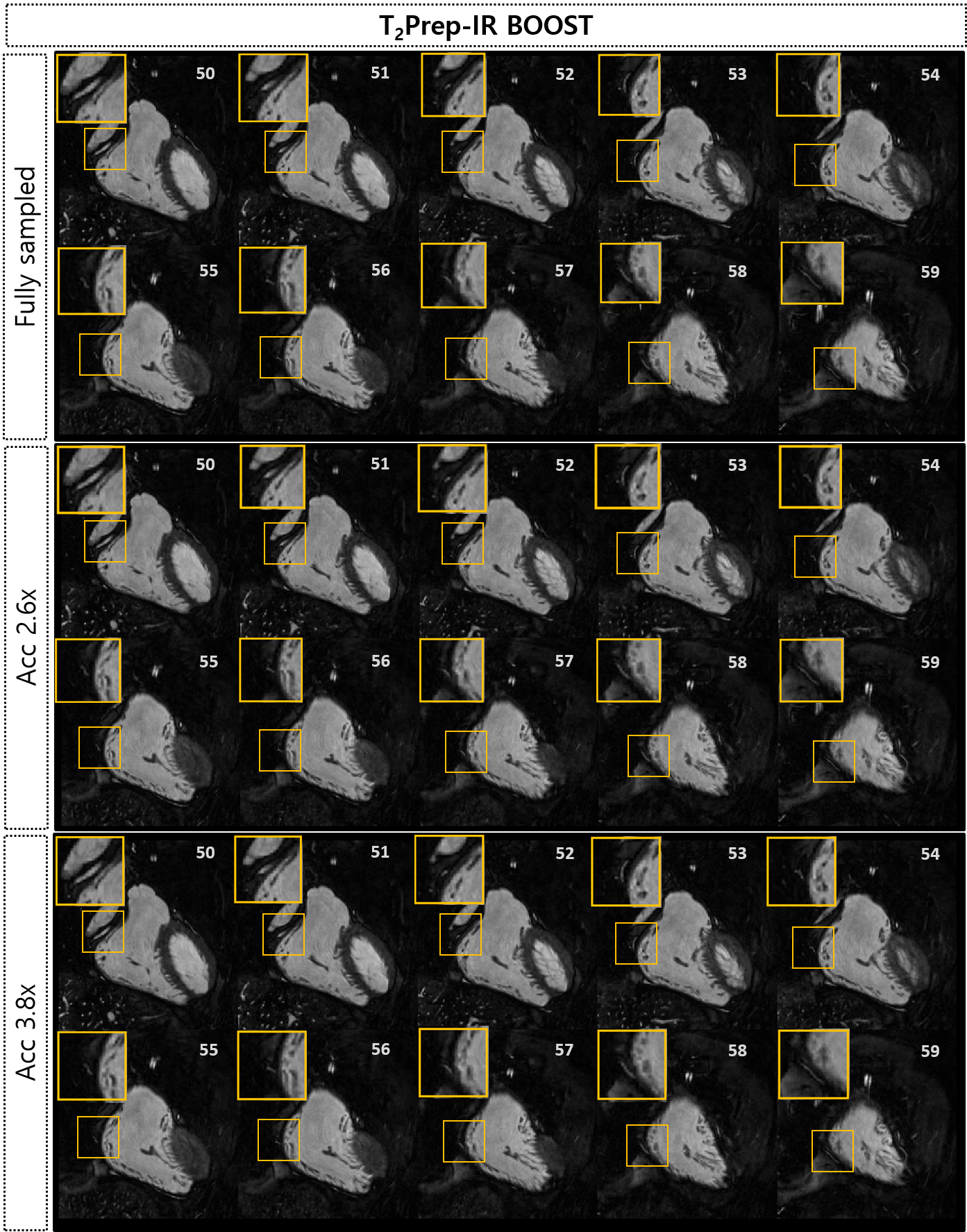

Supplement: Supplementary file 1 — Additional file 1: Fig. S1. Subject 3 reconstructions (coronal views) obtained from fully-sampled, 2.6x and 3.8x accelerated T2Prep-IR BOOST data. The tortuous anatomy of the RCA prevented an appropriate multiplanar reformatting of the mid segment of the vessel. However, the zoomed areas (yellow boxes) of the non-reformatted coronal views show that the mid-RCA was successfully reconstructed from fully-sampled and accelerated BOOST acquisitions using the proposed method. Coronal view numbers are indicated in each subfigure. [file 12968_2020_691_MOESM1_ESM.tif]

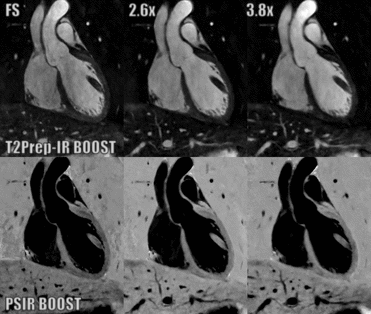

Supplement: Supplementary file 2 — Additional file 2: Fig. S2. Coronal views showing the T2Prep-IR BOOST and PSIR BOOST images reconstructed from fully-sampled, 2.6 x and 3.8x undersampled non-contrast-enhanced BOOST datasets, for a representative healthy subject. Images obtained from accelerated acquisitions have comparable quality to those obtained from fully-sampled acquisitions. The complete heart anatomy and coronary arteries can be clearly visualized in all the images. [file 12968_2020_691_MOESM2_ESM.gif]

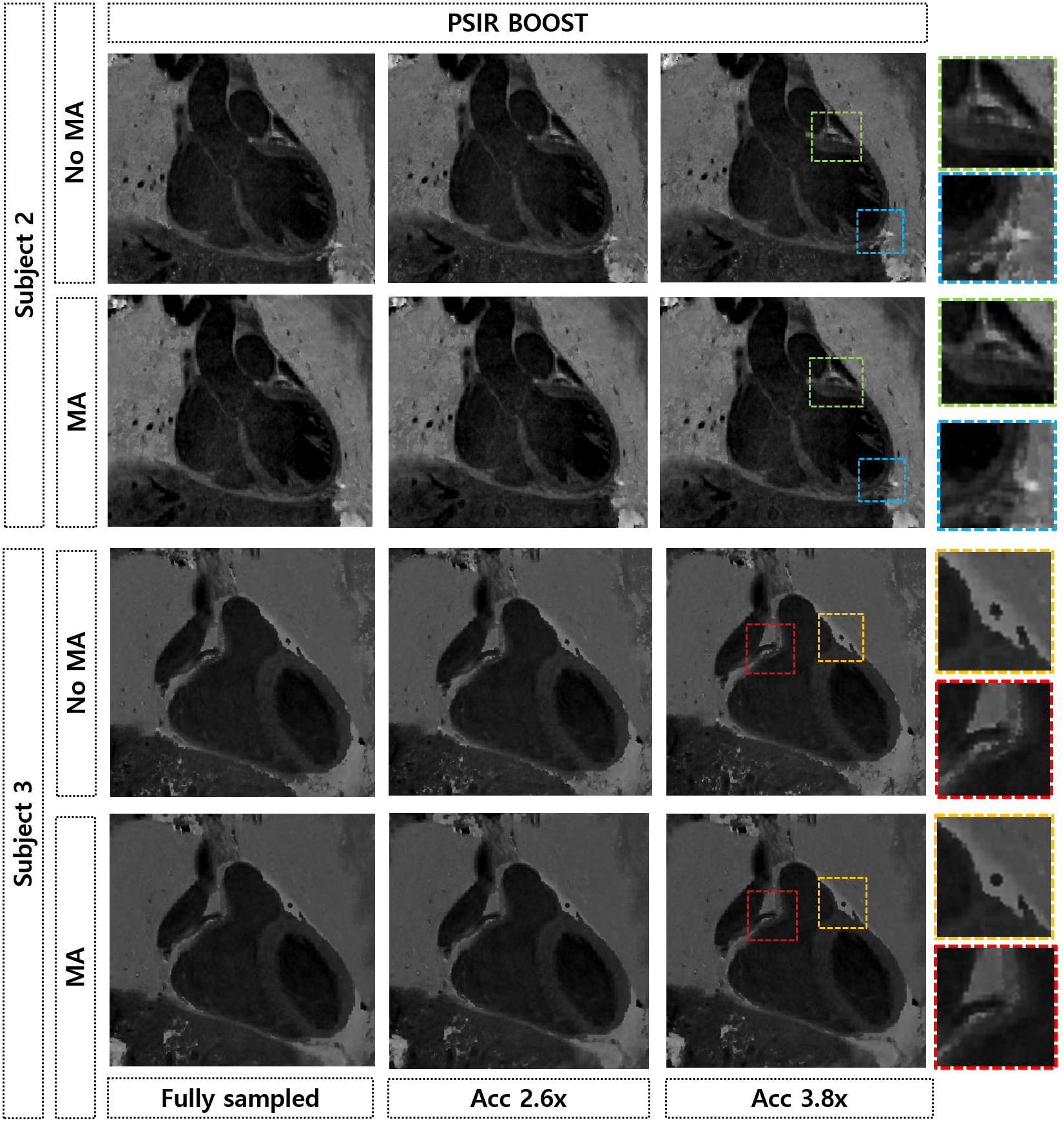

Supplement: Supplementary file 3 — Additional file 3: Fig. S3. 3D black-blood PSIR BOOST coronal views obtained (1st and 3rd rows) without (No MA) and (2nd and 4th rows) with non-rigid motion alignment (NMC) between the T2Prep-IR BOOST and T2Prep BOOST bin images before the PSIR reconstruction, for two representative healthy subjects. The proposed method assumes that the highest quality T2Prep-IR BOOST and T2Prep BOOST bin images are registered. However, there could be some residual non-rigid motion. Hence, non-rigid registration was used to correct for residual non-rigid motion between the T2Prep-IR BOOST and bright-blood T2Prep BOOST bin images. The visual quality of the final 3D black-blood PSIR BOOST image improved slightly when non-rigid motion alignment was used between the T2Prep-IR and T2Prep BOOST images. [file 12968_2020_691_MOESM3_ESM.tif]
